# Electrospun Polydioxanone Loaded With Chloroquine Modulates Template-Induced NET Release and Inflammatory Responses From Human Neutrophils

**DOI:** 10.3389/fbioe.2021.652055

**Published:** 2021-04-27

**Authors:** Allison E. Fetz, Shannon E. Wallace, Gary L. Bowlin

**Affiliations:** Department of Biomedical Engineering, University of Memphis, Memphis, TN, United States

**Keywords:** tissue regeneration, electrospinning, tissue engineering, neutrophils, neutrophil extracellular traps, NETs, chloroquine

## Abstract

The implantation of a biomaterial quickly initiates a tissue repair program initially characterized by a neutrophil influx. During the acute inflammatory response, neutrophils release neutrophil extracellular traps (NETs) and secrete soluble signals to modulate the tissue environment. In this work, we evaluated chloroquine diphosphate, an antimalarial with immunomodulatory and antithrombotic effects, as an electrospun biomaterial additive to regulate neutrophil-mediated inflammation. Electrospinning of polydioxanone was optimized for rapid chloroquine elution within 1 h, and acute neutrophil-biomaterial interactions were evaluated *in vitro* with fresh human peripheral blood neutrophils at 3 and 6 h before quantifying the release of NETs and secretion of inflammatory and regenerative factors. Our results indicate that chloroquine suppresses NET release in a biomaterial surface area–dependent manner at the early time point, whereas it modulates signal secretion at both early and late time points. More specifically, chloroquine elution down-regulates interleukin 8 (IL-8) and matrix metalloproteinase nine secretion while up-regulating hepatocyte growth factor, vascular endothelial growth factor A, and IL-22 secretion, suggesting a potential shift toward a resolving neutrophil phenotype. Our novel repurposing of chloroquine as a biomaterial additive may therefore have synergistic, immunomodulatory effects that are advantageous for biomaterial-guided *in situ* tissue regeneration applications.

## Introduction

Biomaterial-guided *in situ* tissue regeneration utilizes a tissue engineering approach to guide the regeneration of diseased, damaged, or missing tissues (Li et al., 2015). However, compared to exogenous delivery in traditional tissue engineering, *in situ* tissue regeneration relies on the body’s endogenous cells and signals to drive the repair and regeneration processes. Electrospun biomaterials have great potential for guiding *in situ* tissue regeneration because their extracellular matrix (ECM)–mimicking fibers can be fabricated from a variety of biocompatible polymers and modified to suit tissue-specific applications (Lannutti et al., 2007; Kelleher and Vacanti, 2010). Additionally, fabrication of electrospun biomaterials is relatively simple, cost-effective, and easy to scale up for mass production. As such, electrospun templates are an excellent platform for developing biomaterials that guide *in situ* tissue regeneration with the potential for a far-reaching clinical impact.

Independent of location, the implantation of a biomaterial quickly initiates a tissue repair program to restore homeostasis that is initially characterized by an influx of neutrophils (Anderson et al., 2008; Chen and Nuñez, 2010). During the acute inflammatory response, neutrophils condition the microenvironment through multiple mechanisms before recruiting additional immune cells in the tissue repair program. Two of their most significant effector functions include the extrusion of neutrophil extracellular traps (NETs) and the secretion of soluble signals (Brinkmann et al., 2004; Lacy, 2006; Selders et al., 2017). Together, these mechanisms shape the microenvironment for resolution and healing or further perturbations of homeostasis.

NETs are composed of DNA that is complexed with antibacterial neutrophil-derived proteins, including histones, neutrophil elastase, and myeloperoxidase (MPO) (Brinkmann et al., 2004). They are released in response to bacterial signals for the purpose of killing pathogens and are also released in response to inflammatory mediators, such as interleukin 8 (IL-8) and tissue necrosis factor α (TNF-α), activated endothelial cells, and platelets (Gupta et al., 2005, 2010; Clark et al., 2007; Neeli et al., 2008). While they are indispensable for preventing pathogen dissemination, the dysregulated release of NETs is associated with aberrant effects in sterile inflammation due to the localization of noxious cargo that can damage host cells (Gupta et al., 2010; Kaplan and Radic, 2012; Chrysanthopoulou et al., 2014; Gregory et al., 2015). Of particular interest to tissue engineers is the ability of NETs to initiate thrombosis and fibrosis, both of which can be detrimental to functional tissue regeneration (Maugeri et al., 2006; Fuchs et al., 2010; Martinod et al., 2013; Chrysanthopoulou et al., 2014; Gregory et al., 2015; Riehl et al., 2016). In fact, our group has previously shown that NETs are released in response to the surface area–dependent, topographical cues of electrospun polydioxanone (PDO) biomaterials, functioning as a preconditioning event in the tissue repair program (Fetz et al., 2017).

Similar to the release of NETs, neutrophil degranulation and the secretion of soluble signals are meant to neutralize pathogens and initiate the tissue repair program, but they can become dysregulated, leading to tissue damage (Lacy, 2006; Selders et al., 2017). The combination of neutrophil recruitment and tissue damage is typically attributed to the release of proteinases that break down the ECM, such as matrix metalloproteinase 9 (MMP-9), and the release of proinflammatory chemotactic factors, such as IL-8 (Harada et al., 1994; Nagaoka and Hirota, 2000; Martinez et al., 2004; Rayment et al., 2008; Liu et al., 2009). With continual neutrophil recruitment and degranulation, the acute response can develop into a nonresolving, chronic response through a perpetual cycle of recruitment and activation (Martinez et al., 2004; Rayment et al., 2008). Despite these potential deleterious outcomes, neutrophil degranulation and the secretion of signaling molecules have also been shown to be tissue-restorative and proangiogenic (Ardi et al., 2007; Deryugina et al., 2014; Phillipson and Kubes, 2019). Neutrophils secrete vascular endothelial growth factor A (VEGF-A) and hepatocyte growth factor (HGF), both of which support and guide angiogenesis (Taichman et al., 1997; McCourt et al., 2001). Moreover, MMP-9 is also proangiogenic and has been shown to initiate and guide endothelial cell sprouting (Ardi et al., 2007; Christoffersson et al., 2012). Taken together, these data suggest that regulating neutrophil NET release and their secretion of signaling molecules at the onset of the tissue repair program is pertinent for regulating acute neutrophil-driven inflammation (Jhunjhunwala et al., 2015; Herath et al., 2017; Lin et al., 2017; Fetz et al., 2020).

In this work, we evaluated chloroquine diphosphate as an electrospun biomaterial additive to regulate *in vitro* NET release and the secretion of proinflammatory and prohealing mediators from human neutrophils. Chloroquine is a Food and Drug Administration–approved, antimalarial drug that has more recently been investigated as an immunomodulatory and antithrombotic drug for treating rheumatoid arthritis, systemic lupus erythematosus, and cancer (Wozniacka et al., 2006; Manic et al., 2014; Schrezenmeier and Dörner, 2020; Roldan et al., 2020). Furthermore, chloroquine has been shown to inhibit NET formation, indicating its potential benefit as an additive for regulating biomaterial-induced NET release and inflammation (Smith et al., 2014; Boone et al., 2015, 2018; Murthy et al., 2019). Here, we show that electrospun PDO biomaterials loaded with chloroquine modulate template-induced NET release and the inflammatory response from human neutrophils. We found that chloroquine suppresses NET release in a surface area–dependent manner at early time points while modulating proinflammatory and healing signals at both early and late time points. Ultimately, our findings demonstrate a novel repurposing of chloroquine as a template additive for *in situ* tissue engineering that modulates the *in vitro* acute inflammatory response to biomaterials.

## Materials and Methods

### Biomaterial Fabrication

PDO (cat. no. 6100, Bezwada Biomedical, Hillsborough, NJ, United States) was dissolved overnight in 1,1,1,3,3,3-hexafluoro-2-propanol (cat. no. 003409-1KG, Oakwood Chemical, Estill, SC, United States) at varying concentrations (Table 1) to generate biomaterials composed of small and large fibers, previously shown to regulate NET release through their surface area–dependent, topographical cues (Fetz et al., 2017, 2018). Chloroquine diphosphate (cat. no. 0219391910, MP Biomedicals, Solon, OH, United States) was added to the solutions at a concentration of 0.07 mg/mL and dissolved for 1.5 h with gentle agitation before electrospinning. Then, the solutions were loaded into a syringe with a 22.5-gauge blunt needle for the 67 mg/mL PDO solution and an 18-gauge blunt needle for all other solutions and electrospun with optimized parameters (Table 1) to produce small and large fibers as desired (Fetz et al., 2018). Fibers were collected on a 20 × 75 × 5-mm grounded, stainless-steel rectangular mandrel that was rotating 1,250 revolutions/min and translating 6.5 cm/s over 13 cm. Additional higher concentrations of chloroquine were incorporated into the electrospun biomaterials during optimization (Supplementary Methods). For all experiments, 8-mm-diameter discs of the electrospun biomaterials were cut using a biopsy punch (cat. no. P825, Acuderm Inc., Ft. Lauderdale, FL, United States) and stored in a desiccator until use. Prior to cell culture, the biomaterials were irradiated with ultraviolet light at a wavelength of 365 nm using an 8-W lamp (cat. no. EN280L, Spectroline, Westbury, NY, United States) at a working distance of 9.5 cm. The samples were disinfected for 10 min on each side in a sterile, laminar flow hood and kept disinfected until cell culture.

**TABLE 1:** Electrospun biomaterials were fabricated with optimized parameters.

	**Polymer concentration (mg/mL)**	**Chloroquine concentration (mg/mL)**	**Flow rate (mL/h)**	**Airgap distance (cm)**	**Applied voltage (+kV)**
Small fibers	67	0	0.25	13	14
	70	0.07	0.5	12	13
Large fibers	138	0	4.0	28	25
	138	0.07	3.2	28	25

### Biomaterial Characterization

The biomaterials were imaged with a scanning electron microscope, and scanning electron micrographs (SEMs) were analyzed in FibraQuant 1.3 software (nanoTemplate Technologies, LLC) to quantify fiber diameter as previously described (Fetz et al., 2018). Briefly, 150 semiautomated random measurements per SEM were taken to determine the average and corresponding standard deviation for fiber diameter.

### Chloroquine Elution From Biomaterials

The elution of chloroquine from the biomaterials was quantified over the first 24 h using a microplate reader to measure absorbance as described (Lima et al., 2018). The biomaterials (*n* = 4) were placed in a 96-well cell culture plate, and 150 μL of 1 × Hanks buffered salt solution (HBSS, calcium, magnesium, and phenol red free, cat. no. 14175095, Thermo Fisher Scientific, Waltham, MA, United States) was added to each well. After incubating at 37°C for 30 min, 1, 3, 6, and 24 h, the supernatant was removed and refreshed with 150 μL of HBSS. The absorbance of the collected supernatant was read on a SpectraMax i3x Multi-Mode Microplate Reader at 330 nm, and the chloroquine concentration was interpolated from a standard dilution ranging from 333 to 0 μg/mL in HBSS (Supplementary Figure 1). In addition to concentration, the average percent release and standard deviation were calculated for each biomaterial.

### Isolation and Culture of Primary Human Neutrophils

Heparinized whole blood from healthy donors was obtained by venipuncture from Tennessee Blood Services. As purchased or donated samples are not traceable back to the donor, it does not qualify as human subjects research as determined by the University of Memphis Institutional Review Board on November 22, 2016. Neutrophils were then isolated as previously described using Isolymph^®^ density separation (Neeli and Radic, 2013; Fetz et al., 2017, 2018). After isolation, neutrophils were resuspended in HBSS with 10 mM HEPES and 0.2% autologous serum at a concentration of 1 million neutrophils/mL. The disinfected biomaterials (*n* = 3) were placed in a 96-well plate, and 40 μL of the cell culture media was added to each well to hydrate the biomaterials. Negative and positive tissue culture plastic (TCP) wells (*n* = 3) received 30 μL of the cell culture media prior to cell seeding. Subsequently, 100 μL of cell culture media containing 100,000 neutrophils was added to each well followed by 10 μL of heparin (cat. no. H3393, Sigma–Aldrich, St. Louis, MO, United States) at a final concentration of 10 U/mL heparin. Heparin was added to dissociate NET-associated MPO as previously described (Parker et al., 2012; Minden-Birkenmaier et al., 2020). The negative vehicle and positive controls added to TCP wells were 0.15% dimethylsulfoxide in 10 μL of HBSS and 100 nM phorbol 12-myristate 13-acetate (cat. no. P8139, Sigma–Aldrich, St. Louis, MO, United States) in 10 μL of HBSS, respectively. The neutrophils were cultured at 37°C and 5% CO_2_ for 3 and 6 h. Following incubation, the samples were placed on ice for 10 min to inhibit neutrophil stimulation prior to processing. Three experiments were performed with unique donors (male, between 18 and 40 years of age), and the results were pooled for analysis.

### Quantification of NETs and Secreted Signals

Supernatants were collected and assayed using a ProcartaPlex multiplex immunomagnetic assay (cat. no. PPX, Thermo Fisher Scientific, Waltham, MA, United States) on a MAGPIX^®^ microplate reader (Luminex Corporation, Austin, TX, United States). The assayed analytes included angiopoietin, fibroblast growth factor 2, granulocyte colony-stimulating factor (CSF), HGF, IL-1β, IL-1 receptor antagonist, IL-6, IL-8, IL-10, IL-22, monocyte chemoattractant protein 1, MMP-9, MPO, TNF-α, and VEGF-A. To quantify percent NET release, the concentration of MPO was normalized to the concentration of MPO in the positive control at 6 h (Parker et al., 2012; Minden-Birkenmaier et al., 2020).

### Fluorescent Microscopy

Samples were fixed with 10% buffered formalin (cat. no. SF1004, Thermo Fisher Scientific, Waltham, MA, United States) and stained with 5 μM SYTOX orange (cat. no. S34861, Thermo Fisher Scientific, Waltham, MA, United States) and NucBlue^TM^ Fixed Cell ReadyProbes^TM^ Reagent (DAPI, cat. no. R37606, Thermo Fisher Scientific, Waltham, MA, United States) as described (Minden-Birkenmaier et al., 2020). Briefly, samples were sequentially incubated with each stain for 5 min at room temperature. Three washes with 1 × phosphate-buffered saline for 5 min each were performed between each step. Cells and NETs were visualized on an Olympus BX43 fluorescent microscope.

### Statistical Analysis

Statistical significance between fiber diameters was tested with a Kruskal–Wallis and Dunn multiple-comparisons test. All other statistical significance was tested with an analysis of variance and Holm–Sidak multiple-comparisons test. Statistical analyses were performed in Prism version 8.4.3 (GraphPad Software, San Diego, CA, United States) at a significance level of 0.05. Data are reported as mean ± standard deviation.

## Results

### Electrospun PDO Rapidly Elutes Chloroquine

**FIGURE 1:**
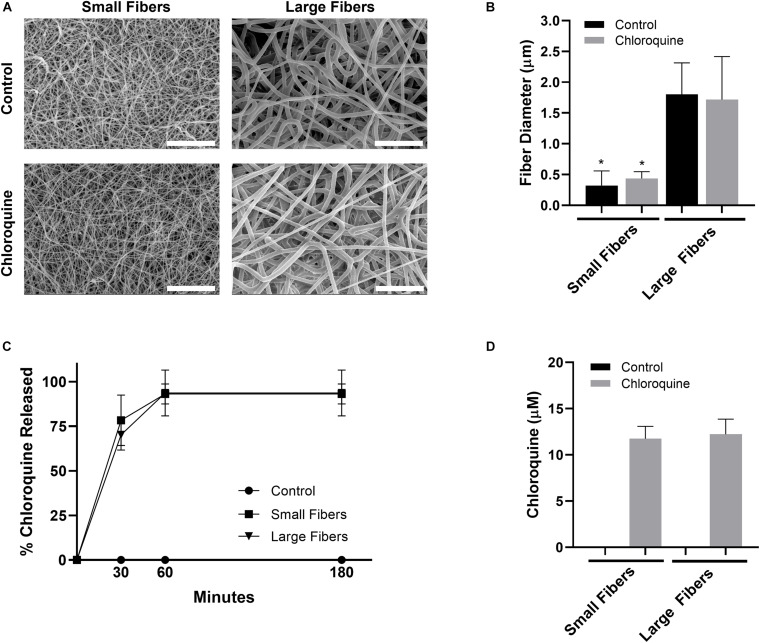
Chloroquine incorporation into electrospun biomaterials results in uniform fibers that rapidly elute the additive. **(A)** Representative SEMs of the control and chloroquine-loaded biomaterials. Micrographs were acquired at 1,000× magnification and scale bars are 30 μm. **(B)** Fiber diameters of the electrospun biomaterials. Measurements (*n* = 150) were taken in FibraQuant 1.3 software. **(C)** Percent chloroquine released from the biomaterials and **(D)** eluted chloroquine concentration at 3 h. There was no increase in concentration after 3 h. See Supplementary Figure 1 for the standard curve used to interpolate concentration (*n* = 4) from absorbance. Graphs show mean ± standard deviation. *p < 0.0001 was determined using a Kruskal–Wallis and Dunn multiple-comparisons test. Raw data are available in Supplementary Spreadsheet 1.

PDO was electrospun to create biomaterials with small and large fibers (Figure 1A). Based on our previous work, the small- and large-fiber biomaterials in this study represent materials that trigger two distinct neutrophil NET responses and two distinct potentials for tissue regeneration (Fetz et al., 2017, 2018). In order to make comparisons independent of fiber size, the polymer concentration was adjusted for the small- and large-fiber biomaterials so that the addition of chloroquine did not alter the resulting fibers (Figure 1B). Any differences in the neutrophil inflammatory response can therefore be attributed to chloroquine elution. Both the small- and large-fiber biomaterials rapidly eluted chloroquine with near 100% elution within the first hour and no detectable increase after 3 h (Figure 1C), which is ideal and desired for targeting the acute neutrophil response during inflammation (Lammermann et al., 2013). The burst elution, driven by segregation of the charged drug to the outer surface of the electrospun fibers (Sun et al., 2008), equated to a concentration of 11.8 ± 1.33 μM and 12.2 ± 1.63 μM chloroquine for the small- and large-fiber biomaterials, respectively (Figure 1D). This elution was optimized by changing chloroquine incorporation during biomaterial fabrication to achieve an eluted concentration near those previously reported in the literature (Smith et al., 2014; Germic et al., 2017). Additional biomaterials were also fabricated to elute higher concentrations of chloroquine (Supplementary Figure 2).

### Chloroquine Elution Inhibits NET Release on Small Fibers

**FIGURE 2:**
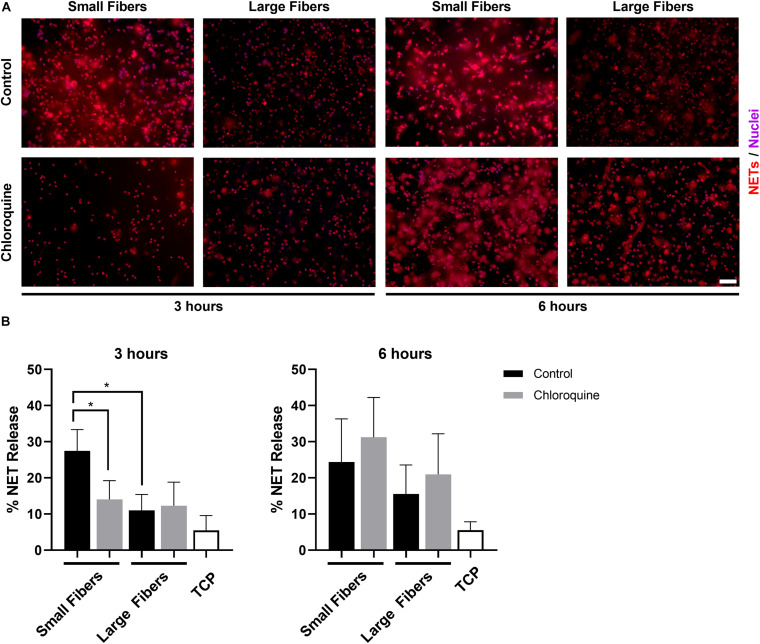
Chloroquine elution inhibits NET release on small fibers but has no effect on large fibers. **(A)** Fluorescent micrographs of neutrophils on the electrospun biomaterials at 3 and 6 h after seeding. Staining of NETs (red) and nuclei (purple) reveals that chloroquine elution from the small fibers attenuates NET formation at the early time point, but not at the late time point. Conversely, chloroquine elution from the large fibers does not modulate NET release. Scale bar is 50 μm. **(B)** Percent NET release at 3 h (left) and 6 h (right) as quantified by the ELISA for NET-disassociated MPO. The quantification of percent NET release (*n* = 3) indicates that chloroquine elution from the small fibers reduces NET release to the level of the large fibers at 3 h with no effect at 6 h. The data represent the mean ± standard deviation of three independent experiments with unique donors. *p < 0.0001 was determined using an analysis of variance (ANOVA) and Holm–Sidak multiple-comparisons test. Raw data are available in Supplementary Spreadsheet 1.

Neutrophils were isolated from the whole blood of healthy donors and seeded on electrospun biomaterials with or without chloroquine to trigger biomaterial-induced NET release. As anticipated, neutrophils had an increased propensity to form NETs on the small fibers compared to the large fibers at 3 h (Figure 2A). More importantly, the elution of chloroquine from the small-fiber biomaterials significantly reduced NET release to the level of the large fibers at 3 h while having no effect upon elution from the large fibers (Figure 2B). By 6 h, the difference between small and large fibers was less pronounced, and increased NET release was observed on both chloroquine-eluting biomaterials, suggesting a temporal, therapeutic window for inhibiting acute NET release (Figure 2B).

### Chloroquine Elution Decreases Inflammatory Signal Secretion

**FIGURE 3:**
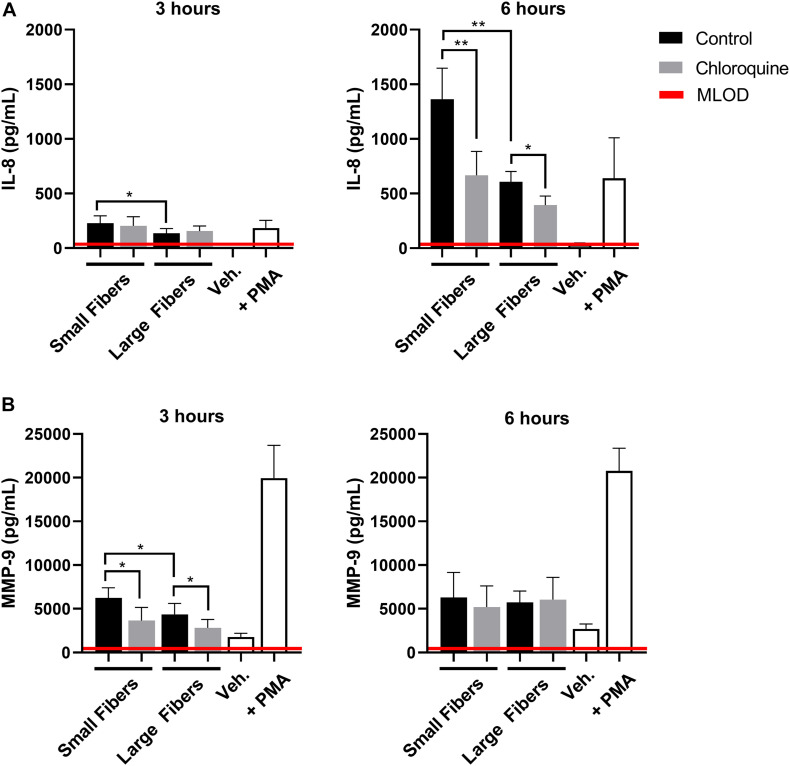
Chloroquine-eluting biomaterials suppress inflammatory IL-8 and MMP-9 secretion from neutrophils. **(A)** IL-8 and **(B)** MMP-9 secretion at 3 h (left) and 6 h (right) after neutrophil seeding. Chloroquine elution down-regulated IL-8 secretion at both time points, whereas it only attenuates the acute secretion of MMP-9. The data (*n* = 3) represent the mean ± standard deviation of three independent experiments with unique donors. *p < 0.05 and **p < 0.0001 were determined using an analysis of variance (ANOVA) and Holm–Sidak multiple-comparisons test. Raw data are available in Supplementary Spreadsheet 1.

Given the documented anti-inflammatory effects, we also evaluated if chloroquine elution would modulate the inflammatory response through the secretion of soluble signals using a multiplexed immunomagnetic assay. Of the assayed inflammatory analytes, only IL-8 and MMP-9 were detected in the supernatant (Figure 3). IL-8 is the archetypal neutrophil chemoattractant secreted by damaged cells as well as neutrophils during an acute inflammatory response. At both 3 and 6 h, IL-8 secretion was significantly greater on the small-fiber biomaterials compared to the large fibers (Figure 3A). As the small fibers appear to up-regulate NET release in a proinflammatory response, it is not surprising that IL-8 secretion mimicked the trends in NET release. However, despite observing a temporal inhibition of acute NET release at 3 h only, the elution of chloroquine from both small and large fibers continued to significantly suppress IL-8 secretion at 6 h. These data suggest there is independent regulation of NET release and IL-8 synthesis and secretion in the context of biomaterial-induced activation, which may be important for reducing aberrant neutrophil recruitment during the tissue repair program (Fujishima et al., 1993; de Oliveira et al., 2013; Degroote et al., 2020). Similar to IL-8, MMP-9 secretion was significantly greater on the small-fiber biomaterials compared to the large fibers with chloroquine elution suppressing secretion at 3 h (Figure 3B). However, unlike IL-8, MMP-9 secretion was near equivalent on all biomaterials by 6 h, suggesting a temporal modulation of MMP-9. As a promiscuous endopeptidase, elevated MMP-9 is correlated with tissue degradation and chronic inflammation, so its acute suppression by chloroquine may prevent triggering a continuum of matrix destruction during the initial inflammatory response (Nagaoka and Hirota, 2000; Rayment et al., 2008; Liu et al., 2009).

### Chloroquine Elution Increases Regenerative Signal Secretion

**FIGURE 4:**
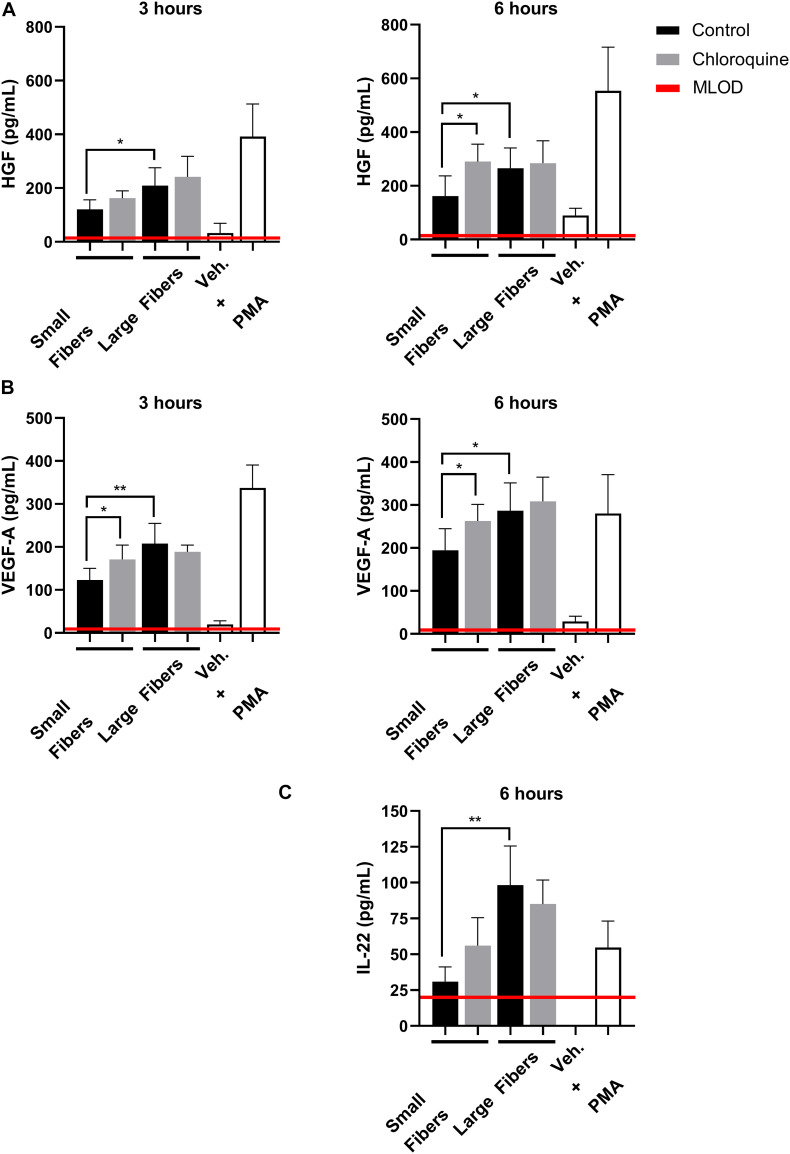
Chloroquine-eluting biomaterials increase regenerative HGF, VEGF-A, and IL-22 secretion from neutrophils. **(A)** HGF and **(B)** VEGF-A were up-regulated with chloroquine elution at 3 h (left) and 6 h (right) after seeding, whereas **(C)** IL-22 was only detectable and up-regulated at 6 h after seeding. The data (*n* = 3) represent the mean ± standard deviation of three independent experiments with unique donors. *p < 0.05 and **p < 0.0001 were determined using an analysis of variance (ANOVA) and Holm–Sidak multiple-comparisons test. Raw data are available in Supplementary Spreadsheet 1.

Although well characterized for its anti-inflammatory effects, chloroquine is not well studied for its potential regenerative effects. Therefore, we also evaluated if chloroquine elution would regulate the secretion of regenerative signals from biomaterial-interacting neutrophils. While several regenerative and anti-inflammatory analytes were assayed, only HGF, VEGF-A, and IL-22 were detected (Figure 4). The secretion of HGF (Figure 4A) and VEGF-A (Figure 4B) followed very similar trends at 3 h, with both having significantly greater secretion on large fibers compared to small fibers, which is the inverse of NET release. Additionally, the elution of chloroquine from the small fibers increased the secretion of HGF and VEGF-A to the level of the large fibers. By 6 h, the trends remained the same with an overall increase in the magnitude of secretion. As they are classic angiogenic signals (Taichman et al., 1997; McCourt et al., 2001; Wislez et al., 2003; Fridlender et al., 2009; He et al., 2016), these data indicate that chloroquine elution may help establish a more regenerative microenvironment around a biomaterial. Likewise, IL-22 secretion followed the same trends as HGF and VEGF-A, but its secretion was not detectable until 6 h, suggesting an absence of readily available stores (Dyring-Andersen et al., 2017). Nonetheless, as a proliferative and proangiogenic signal (Dyring-Andersen et al., 2017; Protopsaltis et al., 2019), the increased IL-22 secretion observed in response to chloroquine elution from the small fibers further suggests that chloroquine modulates the neutrophil phenotype toward healing and regeneration.

## Discussion

Electrospun biomaterials are excellent candidates for guided *in situ* tissue regeneration applications because they have a biomimetic structure that can also function as a drug delivery system to modulate acute inflammation (Kelleher and Vacanti, 2010; Minden-Birkenmaier et al., 2017). In recent years, neutrophils have gained attention as an important part of the acute inflammatory response to a biomaterial and the initiation of the tissue repair program (Fetz et al., 2017, 2020; Selders et al., 2017; Minden-Birkenmaier et al., 2020). Neutrophil recruitment, the release of NETs, and the secretion of soluble mediators can have diametrically opposed effects, leading to the resolution of inflammation and healing or chronic inflammation and fibrosis (Selders et al., 2017). Indeed, seminal work in neutrophil biology has highlighted the phenotypic plasticity of neutrophils and their ability to regulate the tissue environment (Buckley et al., 2006; Fridlender et al., 2009; Horckmans et al., 2016). Given the emphasis on endogenous cells for *in situ* tissue regeneration, regulation of the neutrophil response during acute inflammation is of utmost importance for cell integration and regeneration.

In this work, we developed electrospun PDO biomaterials that elute chloroquine to regulate biomaterial-induced neutrophil activation. PDO is an appealing polymer for electrospun biomaterials because of its biocompatibility, degradation rate, and mechanical properties (Boland et al., 2005; McClure et al., 2009). Additionally, chloroquine is an inexpensive drug historically used to treat malaria that has more recently garnered attention for its immunomodulatory and antithrombotic effects (Wozniacka et al., 2006; Moore et al., 2011; Manic et al., 2014; Schrezenmeier and Dörner, 2020). As a weak base that concentrates in acidic vesicles, it is classified as an inhibitor of lysosomes, lysosomal degradation, and endosomal Toll-like receptor signaling, as well as an inhibitor of autophagy (Sundelin and Terman, 2002; Frustaci et al., 2012; Mauthe et al., 2018). Recently, several groups have shown that chloroquine can be used to inhibit NET release (Smith et al., 2014; Boone et al., 2015, 2018; Germic et al., 2017; Murthy et al., 2019). As it is implicated in tissue fibrosis and thrombosis, NET formation is an appealing pharmacological target, especially in the context of biomaterial-induced NET release (Demers et al., 2012; Chrysanthopoulou et al., 2014; Gregory et al., 2015; Döring et al., 2017; Meng et al., 2017). Our group has shown that neutrophils have an increased propensity to form NETs on the surface of small-fiber electrospun biomaterials, leading to fibrotic encapsulation, whereas large-fiber biomaterials down-regulate NET release and guide tissue integration (Fetz et al., 2017). Jhunjhunwala et al. observed similar outcomes with implanted microcapsules that up-regulated NET release (Jhunjhunwala et al., 2015), thus indicating the need to engineer biomaterials that attenuate NET formation during the acute inflammatory response.

In these experiments, we incorporated chloroquine into the electrospun biomaterials to modulate NET release. At a dose 99.9% lower than the daily oral dose for malaria prophylaxis (Solitro and MacKeigan, 2016; Della Porta et al., 2020), we found that chloroquine eluted from small-fiber biomaterials down-regulated NET release to the level of the large-fiber biomaterials, whereas chloroquine elution from large fibers had no effect on NET release at the early time point. These findings are quite interesting, given that both materials eluted the same concentration of drug with near-identical release profiles. We observed a similar effect upon incorporating Cl-amidine into the electrospun biomaterials, which inhibits the enzyme peptidyl arginine deiminase 4 that is involved in NET formation (Leshner et al., 2012; Fetz et al., 2018). In both cases, these data suggest several mechanisms may be governing the release of NETs on electrospun biomaterials. Additionally, by the later time point, increased NET release was observed on both chloroquine-eluting biomaterials, suggesting a temporal, therapeutic window for inhibiting acute NET release. As the half-life of chloroquine is estimated to be 13–55 days, the observed increase in NET formation at 6 h again suggests that other regulatory mechanisms are involved in biomaterial-induced NET release (Moore et al., 2011; Della Porta et al., 2020). Last, biomaterials that eluted higher concentrations of chloroquine (Supplementary Figure 2) were developed to determine the therapeutic range of NET inhibition by chloroquine. Our data (Supplementary Figure 3) indicate that increasing the chloroquine concentration did not inhibit biomaterial-induced NET release and may have resulted in cytotoxic effects as previously reported (Pliyev and Menshikov, 2012).

Although we and others have shown that chloroquine can inhibit NET release, one group has found that a concentration of chloroquine similar to ours did not inhibit NET formation (Smith et al., 2014; Boone et al., 2015, 2018; Germic et al., 2017; Murthy et al., 2019). When chloroquine was shown to be effective at blocking NET release, neutrophils were stimulated with platelet activating factor, lipopolysaccharide, or our electrospun biomaterials (Smith et al., 2014; Boone et al., 2015). When chloroquine was shown to be ineffective, neutrophils were primed with granulocyte–macrophage CSF and stimulated with C5a, which initiates distinctly different, vital NET release, or the release of mitochondrial NETs (Yousefi et al., 2009; Germic et al., 2017). These data indicate that the therapeutic efficacy of chloroquine for inhibiting NET formation is stimuli dependent and that there may be some overlap in the signaling pathways for biomaterial-induced NET release and other reported triggers of NET release (Farley et al., 2012; Alemán et al., 2016; Khan et al., 2017). Further work is needed to determine the specific signaling pathway involved in biomaterial-induced NET release, but our current data suggest involvement of surface-adsorbed IgG (Fetz et al., 2019).

In addition to inhibiting NETs, chloroquine has been reported to have immunomodulatory effects by altering the secretion of proinflammatory mediators. IL-1β, IL-8, MMP-9, and transforming growth factor β have all been shown to decrease with chloroquine treatment to attenuate inflammation (Delgado et al., 2006; Zhang et al., 2010; Sharma et al., 2017; Fujita et al., 2019). Likewise, in this work, we found that IL-8 and MMP-9 secretions were suppressed on the chloroquine-eluting biomaterials. IL-8 secretion is significantly up-regulated in neutrophils several hours after stimulation before returning to baseline levels, after which monocytes become the major source of IL-8 *in vivo* (Fujishima et al., 1993). Although neutrophils are necessary for tissue healing (Stirling et al., 2009; Horckmans et al., 2016; Lin et al., 2017), these data suggest that suppression of IL-8 by chloroquine at both early and later time points may down-regulate acute inflammation by reducing pernicious neutrophil chemotaxis (Végran et al., 2011). Similar to IL-8, MMP-9 is robustly secreted from neutrophils during acute inflammation and functions to degrade the ECM for enhanced cell motility (Lu et al., 2011; Deryugina et al., 2014). Consequently, MMP-9 can drive both tissue destruction through excessive ECM degradation and the rapid induction of angiogenesis (Ladwig et al., 2002; Ardi et al., 2007, 2009; Rayment et al., 2008; Liu et al., 2009; Hsu et al., 2015). While angiogenesis is paramount for tissue regeneration, elevated levels of MMP-9 could perpetuate inflammation and cyclic matrix destruction, so its suppression by chloroquine may approximate levels more conducive to regeneration, although this remains to be determined.

In conjunction with proinflammatory signals, we also evaluated the potential impact of chloroquine elution on regenerative signals. To our knowledge, no one has yet to explore this aspect of chloroquine’s anti-inflammatory effects. We found that both HGF and VEGF-A secretions were increased with chloroquine elution from the biomaterials. HGF is a proangiogenic growth factor that promotes regeneration and homeostasis while inhibiting chronic inflammation and fibrosis in various tissues (McCourt et al., 2001; Wislez et al., 2003; Matsumoto et al., 2014; He et al., 2016). Similarly, VEGF-A is the canonical angiogenic signal secreted by neutrophils that has also been shown to recruit a proangiogenic subset of neutrophils (Taichman et al., 1997; Fridlender et al., 2009; Christoffersson et al., 2012, 2017). IL-22 closely followed the trends for HGF and VEGF-A, but was only detectable at the later time point, likely because of the time needed to up-regulate synthesis and secretion (Chen et al., 2016). IL-22 has been shown to be both protective and inflammatory, depending on the disease and model (Zindl et al., 2013; Dyring-Andersen et al., 2017; Zenewicz, 2018). However, it was more recently found to support angiogenesis in the tumor microenvironment by inducing endothelial cell proliferation, survival, and chemotaxis (Protopsaltis et al., 2019). Together, the increased secretion of HGF, VEGF-A, and IL-22 on chloroquine-eluting biomaterials suggests a previously unrecognized aspect of chloroquine’s immunomodulatory effects.

Taken together, our novel incorporation of chloroquine into electrospun biomaterials illustrates the potential therapeutic benefit of this drug for biomaterial-guided, *in situ* tissue regeneration applications. Although we are the first to repurpose it as an electrospun template additive for tissue engineering applications, chloroquine has been incorporated into a coating for urine catheters to reduce sterile inflammation by reducing neutrophil necrosis and IL-8 secretion (Puyo et al., 2018). Likewise, our data indicate that local delivery through electrospun PDO biomaterials may down-regulate acute neutrophil-driven inflammation while simultaneously up-regulating their regenerative phenotype. The mechanisms underlying chloroquine’s regulation of NET formation and signal secretion as well as its impact on macrophage phenotype and its *in vivo* efficacy are the subject of further investigations. Additional future work includes evaluation of these biomaterials with platelets and platelet–neutrophil interactions to begin elucidating if chloroquine’s antithrombotic properties are correlated with its inhibition of NET formation (Ouseph et al., 2015; Boone et al., 2018). In conclusion, we have shown that our chloroquine-eluting biomaterials regulate acute neutrophil-driven inflammatory responses *in vitro* by down-regulating NET release and inflammatory signals while up-regulating regenerative signals. These responses may have synergistic effects that are advantageous for biomaterial-guided *in situ* tissue regeneration.

## Data Availability Statement

The original contributions presented in the study are included in the article/Supplementary Material, further inquiries can be directed to the corresponding author/s.

## Ethics Statement

The studies involving human participants were reviewed by the University of Memphis Institutional Review Board. Written informed consent for participation was not required for this study in accordance with the national legislation and the institutional requirements.

## Author Contributions

AF and GB: conceptualization and funding acquisition. AF and SW: methodology and data curation. AF: writing—original draft preparation. AF, SW, and GB: writing—review and editing. GB: supervision and project administration. All authors have read and agreed to the published version of the manuscript.

## Conflict of Interest

The authors declare that the research was conducted in the absence of any commercial or financial relationships that could be construed as a potential conflict of interest.
