# Supplementary material for: Electrospun Polydioxanone Loaded With Chloroquine Modulates Template-Induced NET Release and Inflammatory Responses From Human Neutrophils
Source: Front Bioeng Biotechnol. 2021 Apr 27;9:652055. doi: 10.3389/fbioe.2021.652055 (PMC8111017; doi:10.3389/fbioe.2021.652055)
Supplement: Supplementary file 2 [file Data_Sheet_2.docx]

Supplementary Material

**Supplementary Methods**

*Biomaterial Fabrication*

PDO was dissolved overnight in 1,1,1,3,3,3-hexafluoro-2-propanol (Cat. No. 003409-1KG, Oakwood Chemical, Estill, SC, USA) at varying concentrations (Table S1) to generate biomaterials composed of small and large fibers. Chloroquine diphosphate was added to the solutions at a concentration of 0.07 mg/mL, 0.35 mg/mL and 1.9 mg/mL and dissolved for 1.5 hours with gentle agitation before electrospinning. Following, the solutions were loaded into a syringe with a 22.5-gauge blunt needle for the 67 mg/mL PDO solution and an 18-gauge blunt needle for all other solutions and electrospun with optimized parameters (Table S1). Fibers were collected on a 20 x 750 x 5 mm grounded, stainless steel rectangular mandrel that was rotating 1250 rpm and translating 6.5 cm/s over 13 cm. For all experiments, 8-mm diameter discs of the electrospun biomaterials were cut using a biopsy punch (Cat. No. P825, Acuderm Inc., Fr. Lauderdale, FL, USA) and stored in a desiccator until use. Prior to cell culture, the biomaterials were irradiated with ultraviolet light at a wavelength of 365 nm using an 8 W lamp (Cat. No. EN280L, Spectroline, Westbury, NY, USA) at a working distance of 9.5 cm. The samples were disinfected for 10 minutes on each side in a sterile, laminar flow hood and kept sterile until cell culture.

**Table S1**. Electrospun biomaterials were fabricated with optimized parameters.

|  | ***Polymer Concentration [mg/mL]*** | ***Chloroquine Concentration [mg/mL]*** | ***Flow Rate [mL/h]*** | ***Airgap Distance [cm]*** | ***Applied Voltage [+ kV]*** |
| --- | --- | --- | --- | --- | --- |
| ***Small Fibers*** | *67* | *0* | *0.25* | *13* | *14* |
|  | 70 | 0.07 | 0.5 | 12 | 13 |
|  | 80 | 0.35 | 0.8 | 12 | 16 |
|  | 95 | 1.0 | 0.7 | 12 | 16 |
| ***Large Fibers*** | 138 | 0 | 4.0 | 28 | 25 |
|  | 138 | 0.07 | 3.2 | 28 | 25 |
|  | 150 | 0.35 | 4.2 | 28 | 25 |
|  | 180 | 1.0 | 2.5 | 13 | 15 |

**Supplementary Results**


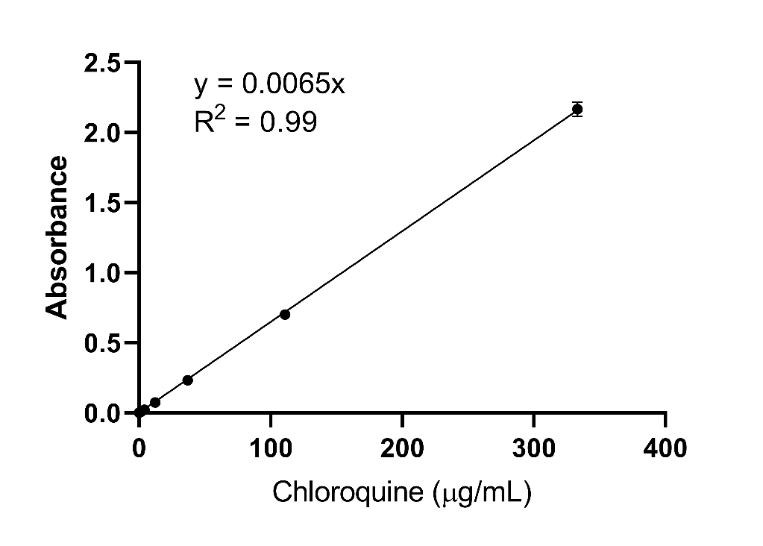


**Supplementary Figure 1**. Chloroquine concentration as determined by absorbance follows a linear trend. The standard dilution of chloroquine was prepared in HBSS and serially diluted by a factor of 3 from 333.3 µg/mL to 1.37 µg/mL. HBSS without chloroquine was included as a background control. Following, 150 µL of the standard dilution and HBSS background control (n = 2) were added to a 96-well plate and absorbance was measured. The resulting data were fit with a line of best fit (y = 0.0065x, R^2^ = 0.99) to create the standard curve for interpolating unknown eluted chloroquine concentration. Raw data is available in Supplementary Spreadsheet 1.


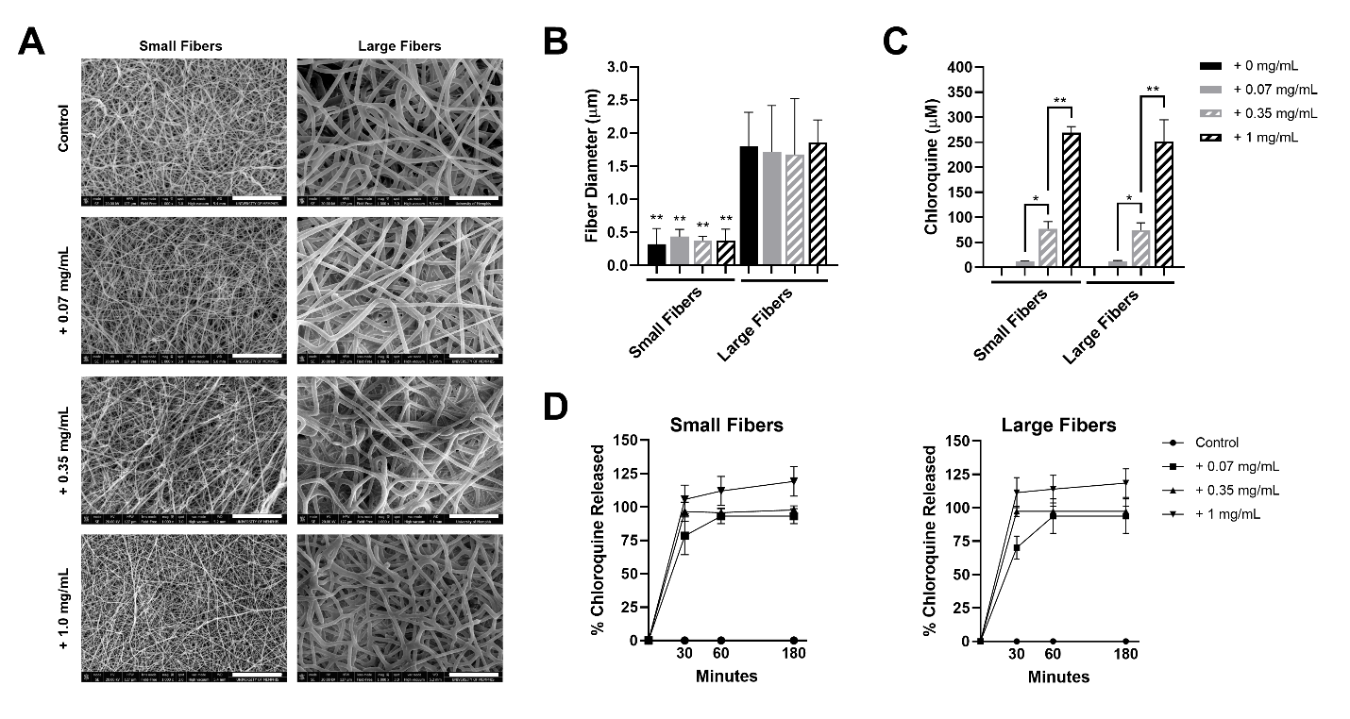


**Supplementary Figure 2**. Chloroquine incorporation into electrospun biomaterials results in uniform fibers that rapidly elute the additive. (A) Representative SEMs of the control and chloroquine-loaded biomaterials. Micrographs were acquired at 1000x magnification and scale bars are 30 µm. (B) Fiber diameters of the electrospun biomaterials. Measurements (n = 150) were taken in FibraQuant 1.3 software. (C) Concentration of eluted chloroquine at 3 hours and (D) percent chloroquine released from the small (left) and large (right) fibers at 3 hours. There was no increase in concentration after 3 hours. See Figure S1 for the standard curve used to interpolate concentration (n = 4) from absorbance. Graphs show mean ± standard deviation. * p < 0.05 and ** p < 0.0001 were determined using a Kruskal Wallis and Dunn’s multiple comparisons test. Raw data is available in Supplementary Spreadsheet 1.


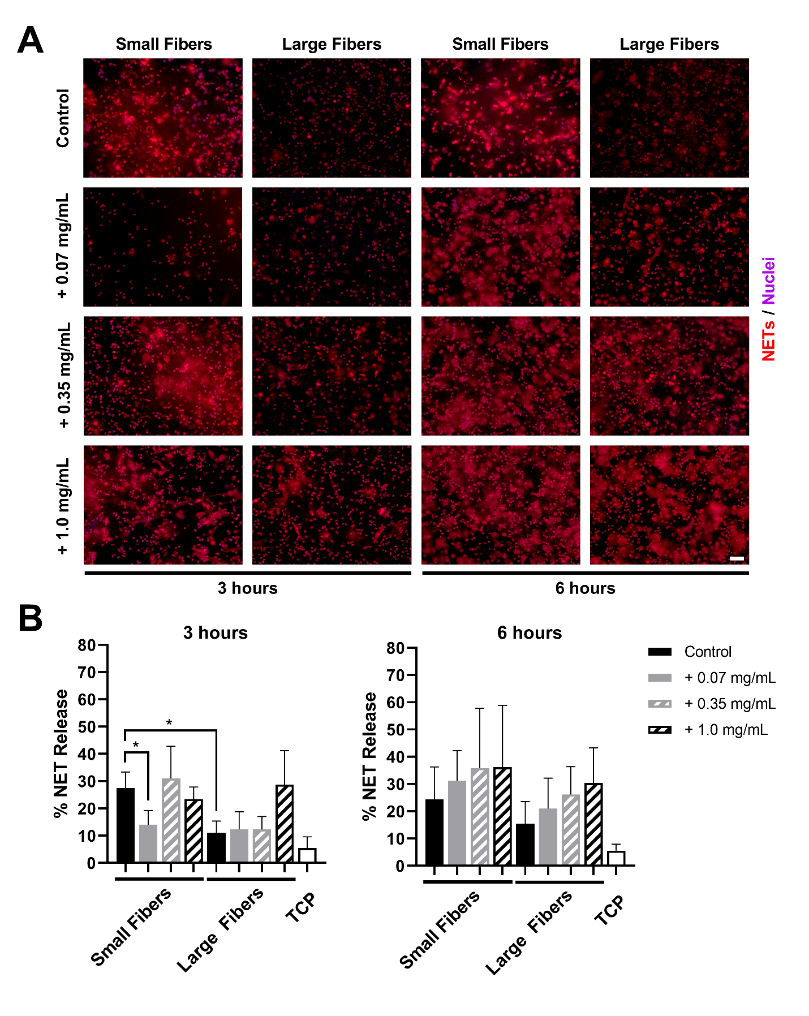


**Supplementary Figure 3.** Chloroquine elution inhibits NET release within a therapeutic window. (A) Fluorescent micrographs of neutrophils on the electrospun biomaterials at 3 and 6 hours after seeding. Staining of NETs (red) and nuclei (purple) reveals that chloroquine elution from the small fibers attenuates NET formation at the early time point at the lowest concentration, but not at the late time point. Conversely, chloroquine elution from the large fibers does not inhibit NET release at any concentration. Scale bar is 50 µm. (B) Percent NET release at 3 (left) and 6 (right) hours as quantified by the ELISA for NET-disassociated MPO. The quantification of percent NET release (n = 3) indicates that chloroquine elution from the small fibers reduces NET release to the level of the large fibers at 3 hours only at the lowest dose. At 6 hours, both small and large fibers eluting higher concentrations of chloroquine result in an apparent increase in NET release, suggesting a therapeutic window at the lowest chloroquine concentration and potential cytotoxic effects at higher concentrations [71]. The data represent the mean ± standard deviation of three independent experiments with unique donors. * p < 0.0001 was determined using an ANOVA and Holm-Sidak’s multiple comparisons test. Raw data is available in Supplementary Spreadsheet 1.
